# Identifying effective components for mobile health behaviour change interventions for smoking cessation and service uptake: protocol of a systematic review and planned meta-analysis

**DOI:** 10.1186/s13643-017-0591-7

**Published:** 2017-10-06

**Authors:** Pritaporn Kingkaew, Liz Glidewell, Rebecca Walwyn, Hamish Fraser, Jeremy C. Wyatt

**Affiliations:** 1 Leeds Institute of Health Sciences, University of Leeds, Worsley Building, Clarendon Way, Leeds, UK; 2 Leeds Institute of Clinical Trials Research, University of Leeds, Worsley Building, Clarendon Way, Leeds, UK; 3 Wessex Institute of Health and Research, Faculty of Medicine, University of Southampton, Southampton, UK; 4 Health Intervention and Technology Assessment Program, Ministry of Public Health, Nonthaburi, Thailand

**Keywords:** Complex interventions, Tobacco cessation, Mobile phones, Mobile health, Behaviour change techniques, Theory-based interventions, Systematic review, Meta-analysis, Protocol

## Abstract

**Background:**

Mobile health (mHealth) interventions for smoking cessation have been shown to be associated with an increase in effectiveness. However, interventions using mobile phones to change people’s behaviour are often perceived as complex interventions, and the interactions between several components within them may affect the outcome. Therefore, it is important to understand how we can improve the design of mHealth interventions using mobile phones as a medium to deliver services.

**Methods:**

Randomised controlled trials (RCTs) of mHealth interventions to support smoking cessation or uptake of smoking cessation services for smokers will be included in this systematic review. A search will be performed by searching MEDLINE, MEDLINE(R) In-Process & Other Non-Indexed Citations, EMBASE, PsycINFO, Web of Science, and CINAHL. A search for new publications will be conducted 3 months prior to submission for publication as mHealth is an emerging area of research.

A random-effects meta-analysis model will be used to summarise the effectiveness of mHealth interventions. The risk ratio will be used for the primary outcome, self-reported or verified smoking abstinence, and any binary outcomes for uptake of smoking cessation services. The standardised mean difference using Hedges’ *g* will be reported for continuous data. Heterogeneity will be assessed using *I* ^2^ statistics.

Where feasible, meta-regression analysis using random-effects multilevel modelling will be conducted to examine the association of pre-specified characteristics (covariates) at the study level with the effectiveness of interventions. Publication bias will be explored using Egger’s test for continuous outcomes and Harbord and Peters tests for dichotomous outcomes. The funnel plot will be used to evaluate the presence of publication bias. The Cochrane Risk of Bias Tool will be used to assess differences in risks of bias.

**Discussion:**

The results of this systematic review will provide future research with a foundation for designing and evaluating complex interventions that use mobile phones as a platform to deliver behaviour change techniques.

**Systematic review registration:**

PROSPERO CRD42016026918.

**Electronic supplementary material:**

The online version of this article (10.1186/s13643-017-0591-7) contains supplementary material, which is available to authorized users.

## Background

Tobacco smoking is widely recognised as one of the leading threats to population health. Tobacco use is an avoidable behavioural risk factor that causes many diseases such as cardiovascular disease, respiratory diseases, and several cancers and neoplasms. It is estimated that there are one billion adult smokers in the world [1], and around 10% of deaths globally per year will be attributable to tobacco use [2].

Mobile health (mHealth) is an emerging area of research with various fields for application, one of which is for behaviour change. mHealth interventions have been shown to be associated with better behaviour, especially in the smoking cessation field [3–5]. Results from published meta-analyses suggest that mobile phone-based interventions for smoking cessation are beneficial compared to controls without mobile phone interventions [3–8]. mHealth can also be used as an intervention to add onto existing smoking cessation services by identifying more people to engage in smoking cessation services. However, these existing systematic reviews have not examined whether mobile phone interventions can increase the proportion of participants deciding to engage with smoking cessation services, and only consider smoking cessation outcomes.

mHealth for behaviour change interventions are often perceived as complex interventions because they contain several interacting components and have several dimensions of complexity. However, defining intervention components can be challenging due to the nature of complex interventions [9]. The typical question of whether it works may no longer be sufficient but understanding how it works is also crucial [10]. Several components within these complex interventions may affect the outcome such as mode of delivery [11], duration and intensity of the intervention [4], and tailored functionality [5, 12].

The use of theory to design and develop complex interventions is recommended by the UK Medical Research Council (MRC) [13, 14]. Theory-based behaviour change interventions have been used in many mHealth interventions. Theory can be applied in various approaches such as recruiting participants, designing an intervention, and planning for evaluation [15]. Though the use of theory—classified very simply as yes or no—was not found to be associated with the effect size of text messaging-based health promotion interventions [5], it was found to differ where extensive use of theory was associated with intervention effect size of internet-based behaviour change interventions [11].

Behaviour change techniques (BCTs) are identifiable ‘active ingredients’ of behaviour change interventions derived from behaviour change theory [16]. A taxonomy of BCTs specific to smoking cessation has been developed [17]. It has been used to identify behaviour change techniques that are associated with higher effect size such as action planning, self-monitoring, social support, and advice on weight control [18]. The ability to define and recognise such BCTs could help researchers improve knowledge regarding effective behaviour change interventions. The latest hierarchically structured taxonomy of 93 behaviour change techniques was developed to serve as a more reliable and systematic specification of BCTs for generic use [16].

Defining intervention components can be challenging. A Template for Intervention Description and Replication (TIDieR) checklist can be used to assess the quality of reporting of complex interventions [19]. It can also help future research identify the important items to be reported for complex interventions. Therefore, the use of the TIDieR checklist would allow readers to systematically assess the quality of the report but would also allow them to systematically synthesise the components of complex interventions.

A lack of evidence on effective components of mHealth interventions for smoking cessation and a lack of outcomes reported in terms of smoking cessation service uptake limit the future design of these interventions. Therefore, to understand how we can improve the design of mHealth interventions as a medium to deliver smoking cessation services, the review questions for this study include: (1) what is the effectiveness of mHealth behaviour change interventions for smoking cessation?; (2) what is the effectiveness of mHealth behaviour change interventions for uptake of smoking cessation services?; (3) which components of mHealth behaviour change interventions are associated with improvements in smoking cessation rates?; and (4) which components of mHealth behaviour change interventions are associated with improvements in uptake of smoking cessation services? The results from this review should be beneficial to researchers planning for and developing complex mHealth interventions.

## Methods

### Protocol and study registration

The Preferred Reporting Items for Systematic Review and Meta-Analysis Protocols (PRISMA-P) 2015 [20] and its supporting paper [21] were used as a guide to develop this protocol. See Additional file 1 for the PRISMA-P checklist. The protocol of this systematic review was registered on the PROSPERO International prospective register of systematic reviews (PROSPERO CRD42016026918).

### Eligibility criteria

There will be no restrictions for settings and countries of origin. Studies will be selected for analysis according to the following criteria.

#### Participants

Participants are all smokers, including those who intend to quit smoking and those who do not intend to quit smoking, from any sources or settings.

#### Interventions

Interventions aimed at smoking cessation that are delivered through or in combination with mobile phones via short messaging services (SMS), multimedia messaging services (MMS), phone calls, interactive voice responses (IVR), email, web browsers, social media, and apps will be included. The use of mobile phones only for research design facilitation or for data collection purposes will be excluded. Interventions aimed at preventing new smokers will also be excluded from this study.

#### Comparators

Comparators include no interventions or usual care or alternative mHealth interventions.

#### Outcomes

Primary outcome includes biochemically verified or self-reported smoking abstinence at any follow-up period (e.g. 1, 3, 6 months or longer). Secondary outcomes include only biochemically verified smoking abstinence at any follow-up period and reported uptake of smoking cessation services at any follow-up period. Reported uptake of smoking cessation services includes behaviour-related outcomes such as the number of smoking cessation services attendance.

#### Study designs

Randomised controlled trials (RCTs) will be included in the review. Controlled clinical trials (CCTs), controlled before-after (CBA) studies, and studies that do not have a control group such as cross-sectional studies, case series, and case reports will be excluded from the review.

### Search strategy

A search will be performed by searching Ovid MEDLINE(R), Ovid MEDLINE(R) In-Process & Other Non-Indexed Citations, EMBASE, PsycINFO, Web of Science, CINAHL (EbscoHOST), and LILACS. Text words and, if available, subject heading terms for mobile phone, text messaging, mobile application, interactive voice response, email, internet, web browser, social media, smoking cessation, tobacco use, smoking, tobacco-use disorder, smoking behaviour, and randomised control trial will be used. To ensure the quality of the search strategy, it was peer reviewed by an information specialist using the Peer Review for Electronic Search Strategies (PRESS) template [22, 23]. Additional file 2 shows the search strategy from Ovid MEDLINE(R). A review of grey literature will be conducted through a search from Open Grey and WorldCat. Reference lists of included studies will be screened for relevant studies. An update search will commence in August 2017 to check for new publications.

### Study selection

All studies retrieved using the search strategy will be managed using EndNote X7 reference (Thomson Reuters (Scientific) LLC, New York). All duplicates will be screened within EndNote X7 reference. Titles and abstracts of studies will be screened by one reviewer (PK) using the eligibility form (see Additional file 3). A random sample of 20% of all abstracts will be independently checked by two reviewers (PK and LG). Reliability and agreement rate will be tested prior to full review. The full text of included studies will then be retrieved and assessed for eligibility by one reviewer (PK). A random sample of 20% of the full texts will be independently checked by two reviewers (PK and LG). Disagreements between the review authors over the inclusion of the sample of 20% of the full texts will be resolved by discussion. Cohen’s kappa (Cohen 1960) will be used to report the degree of agreement.

### Data collection and data extraction

Full text of eligible studies will be explored for information related to the development of the interventions. If it states elsewhere, such as the study protocol, descriptive and qualitative study, additional references will be included for data extraction purposes only. Separate notation of sources of information will be identified. Information to be extracted are presented in Table 1. Coding will be conducted for both intervention groups and comparator groups (control, usual care, and alternate interventions).

**Table 1:** Information to be extracted from eligible studies

Lists	Information to be extracted
Publication details	First author name, year of publication
Study setting	Country and source of participants, e.g. primary/secondary care, vpharmacy, advertisement
Duration of study and follow up	Reported information on the duration of study and follow up for each outcome
Year of study	Year of study
Participant demographics	Participant mean age, percentage of current smokers, average number of cigarettes per day
Sample size	Sample sizes included in the analysis
Perceived barriers	Identified barriers of behaviour change prior to intervention design
The use of theory to design intervention	No theory used/the use of theory to inform interventions/the use of theory to classify participants/the use of theory to tailor interventions according to participants 19 Theory Coding Scheme (when > 50 studies are included)
Behaviour change techniques	Coding scheme for behaviour change technique using BCTTv1 [16]
Mobile functionality	SMS, MMS, email, phone call, internet, apps, IVR
Tailored design	No tailoring function (fixed intervention)/personalisation based on participant characteristics or personal preferences/tailored to participant needs
Communication pathway	One-way/two-way/interactive communication
Description of technology engagement	Detailed description of any form of measurement for engagement in technology, e.g. automated monitoring of the users’ interactions with the system, etc.
Description of control and intervention groups	Detailed description of control and intervention groups
Outcomes measured	Self-reported smoking abstinence Verified smoking abstinence Uptake of smoking cessation services, e.g. the number of service attendance
Reference of additional information	Additional information elsewhere that is related to the intervention design

#### Coding procedures for behaviour change techniques

Generic behaviour change technique taxonomy will be used in this study [16]. Standardising the coding of the behaviour change techniques requires experience [24]. Therefore, prior to coding, training will be carried out using the BCTTv1 training online. Subsequently, the trained coders (LG and PK) will independently identify the BCTs from 20% of all included studies. Discrepancies regarding the BCT coding will be resolved through discussion. BCTs will then be mapped to the COM-B system where the interaction of capability, opportunity, and motivation leads to behaviour change [25].

### Risk of bias and quality assessment

Studies that are included for data synthesis will be assessed for risks of bias using the Cochrane Risk of Bias Tool [26]. A random sample of 20% of the full texts will be independently checked by two reviewers (PK and RW). Disagreements between the review authors over the risk of bias will be resolved through discussion. A sensitivity analysis to exclude studies that are shown to have high risk of bias will be conducted. In addition, all included studies will be assessed using the Template for Intervention Description and Replication (TIDieR) checklist—used to assess the quality of reporting of complex interventions—to provide more information about complex interventions [19]. We will report this as a percentage of studies that are reported in each criterion.

### Data synthesis

A descriptive summary table of the included studies will be summarised (see Table 1). When there is a sufficient number of studies (*k* > 10) reporting for similar outcomes [27], meta-analysis will be used to estimate the pooled treatment effect. To determine the effect size, the risk ratio (RR) will be used for the primary outcome, self-reporting, or verified smoking abstinence (e.g., smoking status: yes/no). The standardised mean difference, using Hedges’ *g* [28], will be used for continuous outcome measurements comparing between the treatment and control groups (e.g., increase number of smoking cessation service attendance). All statistical analyses will be undertaken using Stata 14 software [29].

A meta-analysis (the *metan* command) will be conducted using random-effects model (inverse-variance methods and DerSimonian and Laird methods of moment estimator), with 95% confidence intervals and significance level at 5% [30]. Random-effects model recognises within-study variance and between-study variance. The nature of the studies included in this systematic review is likely to be different (intervention and patient population), and the heterogeneity between studies is expected. Consistent with this assumption, random-effects model will be used to estimate a pooled treatment effect. Heterogeneity between studies will be assessed using *I* ^2^ statistics. When *I* ^2^ is over 50% (moderate heterogeneity), heterogeneity will be addressed through meta-regression and sensitivity analyses [31, 32].

In order to address any publication bias, a funnel plot of log odds ratio against standard error of log odds will be conducted for each outcome. Egger’s test will be used to test for asymmetry for continuous outcomes [33] whereas Peters [34] and Harbord [35] tests will be used for binary data. Peters and Harbord tests were proposed to be used to avoid the mathematical association between the log odds ratio and its standard error (false-positive test results) that occurs from Egger’s test [26]. The fixed- and random-effects estimates of the intervention effect will be compared if there are any small-study effects. When there is an evidence of small-study effects on the pooled effect, sensitivity analysis based on selection models proposed by Terrin et al. [36] will be used to estimate a pooled effect adjusted for selection bias [37]. This method is recommended over the trim-and-fill method as it provided better performance in simulation study.

### Meta-regression

A meta-regression analysis will be conducted in order to define which components of mHealth behaviour change interventions are associated with improvements in smoking cessation rates and uptake of smoking cessation services when there is at least 10 studies [27]. Pre-specified covariates are recommended in order to avoid false-positive conclusions. Covariates that will be fitted in univariate and multivariate analyses include the duration of intervention, the use of theory to design the intervention, behaviour change techniques, mobile functionality, tailored design, and communication pathway.

Two approaches for the meta-regression analysis will be used. The first approach assumes all control interventions across studies are the same (the *metareg* command). This first approach will regress only covariates (characteristics) from intervention arms using random-effects meta-regression. The second approach will consider the characteristics of controls in addition. A multilevel logistic regression for repeated measures, allowing for different follow-up times, will be used to estimate the effect of interventions towards binary outcomes, including the primary outcome—self-reporting smoking abstinence (the *meqrlogit* command). If available, a multilevel regression analysis will be used to estimate the effects of interventions for other continuous outcomes.

#### Sub-group analysis

Sub-group analyses will explore when studies with high risk of bias are excluded. Sub-group analysis will also be conducted to consider characteristics of intervention and control groups specified in Table 1, including the use of theory to design intervention, behaviour change techniques, mobile functionality, tailored design, and communication pathway.

## Discussion

This protocol states the plan for a systematic review and meta-analysis of mobile health behaviour change interventions for smoking cessation and service uptakes. While there are a number of meta-analysis and meta-regression studies for smoking cessation, the majority of mHealth systematic reviews for smoking cessation focuses more on the technology components rather than behaviour change components except for a systematic review conducted by Free et al. [3]. However, this review only showed the descriptive results of the number of BCTs used in each study [3]. To our knowledge, there is a similar planned systematic review and meta-analysis of behaviour change interventions to support smoking cessation [38]. However, our study will be focusing on the technology aspects as well as behaviour change techniques and will include studies with all follow-up periods. While de Bruin [38] plans to extract information regarding BCTs from primary research groups, the BCTs identified from research groups can be subjected to bias due to retrospective data collection.

A potential limitation of this study is that the outcomes for smoking cessation service uptakes are still unknown. This may include a wide range of indicators, and therefore a meta-regression may not be able to be conducted where the number of papers is a significant factor for analysis. As such, descriptive data synthesis is expected in this case. We hope that this study will help provide a platform for future designs of smoking cessation using mobile phones as a medium.

## Additional files

Additional file 1: PRISMA-P checklist. (DOCX 15 kb)

Additional file 2: Search strategy. (DOCX 15 kb)

Additional file 3: Study eligibility form. (DOCX 29 kb)

## Abbreviations

BCTs: Behaviour change techniques
BCTTv1: Behaviour change technique taxonomy version 1
CBA: Controlled before-after study
CCTs: Controlled clinical trials
IVR: Interactive voice responses
mHealth: Mobile health
MMS: Multimedia messaging services
MRC: Medical Research Council
PRESS: Peer Review for Electronic Search Strategies
PRISMA-P: Preferred Reporting Items for Systematic Review and Meta-Analysis Protocols
RCTs: Randomised controlled trials
SMS: Short messaging services
TIDieR: Template for Intervention Description and Replication
