# Supplementary material for: Identifying effective components for mobile health behaviour change interventions for smoking cessation and service uptake: protocol of a systematic review and planned meta-analysis
Source: Syst Rev. 2017 Oct 6;6:193. doi: 10.1186/s13643-017-0591-7 (PMC6389191; doi:10.1186/s13643-017-0591-7)
Supplement: Supplementary file 2 — Search strategy. (DOCX 15 kb) [file 13643_2017_591_MOESM2_ESM.docx]

# Additional file 2 Search Strategy

**Database:** Ovid MEDLINE(R)

----------------------------------------------------------------------------------------------------------------

1 exp Cell Phones/

2 (cell$ adj3 phone$).tw.

3 (cell$ adj3 telephone$).tw.

4 (mobile adj3 phone$).tw.

5 (mobile adj3 telephone$).tw.

6 smartphone$.tw.

7 smart-phone$.tw.

8 exp text messaging/

9 SMS.tw.

10 (short adj messag$).tw.

11 (text adj messag$).tw.

12 texting.tw.

13 (multimedia adj messag$).tw.

14 (messag$ adj service$).tw.

15 interactive voice response.tw.

16 (interactive adj voice).tw.

17 (voice adj response).tw.

18 exp Electronic Mail/

19 email?.tw.

20 e-mail?.tw.

21 (electronic adj mail?).tw.

22 exp Internet/

23 internet-based.tw.

24 exp Web Browser/

25 web-based.tw.

26 exp Social Media/

27 (social adj media).tw.

28 (digital adj media).tw.

29 exp mobile application/

30 (mobile adj3 app?).tw.

31 (mobile adj3 application?).tw.

32 (phone$ adj3 app?).tw.

33 (phone$ adj3 application?).tw.

34 (smartphone$ adj3 app?).tw.

35 (smartphone$ adj3 application?).tw.

36 (smart-phone$ adj3 app?).tw.

37 (smart-phone$ adj3 application?).tw.

38 1 or 2 or 3 or 4 or 5 or 6 or 7 or 8 or 9 or 10 or 11 or 12 or 13 or 14 or 15 or 16 or 17 or 18 or 19 or 20 or 21 or 22 or 23 or 24 or 25 or 26 or 27 or 28 or 29 or 30 or 31 or 32 or 33 or 34 or 35 or 36 or 37

39 exp Smoking Cessation/

40 exp "tobacco use"/

41 exp Tobacco Products/

42 exp smoking/

43 exp "Tobacco Use Disorder"/

44 (smok$ adj1 cessation$).tw.

45 (smok$ adj1 stop*).tw.

46 (smok$ adj1 quit*).tw.

47 (smok$ adj1 giv* up).tw.

48 (tobacco adj1 cessation$).tw.

49 (smok$ adj1 behavio$).tw.

50 smoking.tw.

51 tobacco.tw.

52 cigar?.tw.

53 cigarette?.tw.

54 39 or 40 or 41 or 42 or 43 or 44 or 45 or 46 or 47 or 48 or 49 or 50 or 51 or 52 or 53

55 randomized controlled trials as topic/

56 randomized controlled trial/

57 (controlled adj trial$).tw.

58 (random$ adj trial$).tw.

59 ((singl$ or doubl$ or treb$ or tripl$) adj (blind$3 or mask$3)).tw.

60 (allocated adj2 random$).tw.

61 55 or 56 or 57 or 58 or 59 or 60

62 case report.tw.

63 letter/

64 historical article/

65 animals/ not (human/ and animals/)

66 62 or 63 or 64 or 65

67 61 not 66

68 38 and 54 and 67
