# Supplementary material for: Identifying effective components for mobile health behaviour change interventions for smoking cessation and service uptake: protocol of a systematic review and planned meta-analysis
Source: Syst Rev. 2017 Oct 6;6:193. doi: 10.1186/s13643-017-0591-7 (PMC6389191; doi:10.1186/s13643-017-0591-7)
Supplement: Supplementary file 3 — Study eligibility form. (DOCX 29 kb) [file 13643_2017_591_MOESM3_ESM.docx]

# Additional file 3 Study eligibility form

| **Factors** | **Assessment** | **Comments** |
| --- | --- | --- |
| **Type of study** | | |
| 1. Is the study described as a randomized controlled trial (RCT)? | Yes Unclear No    Exclude |  |
| **Participants** | | |
| 1. Did studies include smokers who do not intend or do intend to quit smoking, from any sources or settings | Yes Unclear No    Exclude |  |
| **Interventions** | | |
| 1. Were interventions delivered through mobile phones or with mobile phone? Delivery mode can be text messaging, mobile apps, interactive voice responses, email, internet, web browser, and social media. | Yes Unclear No    Exclude |  |
| 1. Were interventions aimed at increasing smoking cessation rates or uptake of smoking cessation services? | Yes Unclear No    Exclude |  |
| 1. Were comparison groups are either no interventions (usual care or no mobile phone intervention) or alternative mobile phone interventions? | Yes Unclear No    Exclude |  |
| **Outcomes** | | |
| 1. Did the study report smoking cessation rates or any kinds of smoking cessation service uptake? | Yes Unclear No    Exclude |  |
| FINAL DECISION |  |  |
